# Bridging the Gap: using an interrupted time series design to evaluate systems reform addressing refugee maternal and child health inequalities

**DOI:** 10.1186/s13012-015-0251-z

**Published:** 2015-04-30

**Authors:** Jane Yelland, Elisha Riggs, Josef Szwarc, Sue Casey, Wendy Dawson, Dannielle Vanpraag, Chris East, Euan Wallace, Glyn Teale, Bernie Harrison, Pauline Petschel, John Furler, Sharon Goldfeld, Fiona Mensah, Mary Anne Biro, Sue Willey, I-Hao Cheng, Rhonda Small, Stephanie Brown

**Affiliations:** Healthy Mothers Healthy Families Research Group, Murdoch Children’s Research Institute, Parkville, 3052, VIC Australia; Department of General Practice and Primary Health Care Academic Unit, University of Melbourne, Parkville, VIC Australia; Victorian Foundation for Survivors of Torture, Brunswick, VIC Australia; Monash Women’s Maternity Services, Monash Health, Clayton, VIC Australia; School of Nursing and Midwifery, Monash University, Clayton, VIC Australia; The Ritchie Centre, Monash University, Clayton, VIC Australia; Department of Obstetrics and Gynaecology, Monash University, Clayton, VIC Australia; Women’s and Children’s Services, Western Health, Sunshine, VIC Australia; Department Obstetrics and Gynaecology, University of Melbourne, Parkville, VIC Australia; Maternal and Child Health, City of Greater Dandenong, Dandenong, VIC Australia; Maternal and Child Health, City of Wyndham, Wyndham, VIC Australia; Centre for Community Child Health, Murdoch Children’s Research Institute, Royal Children’s Hospital, Parkville, VIC Australia; Department of Paediatrics, University of Melbourne, Parkville, VIC Australia; Clinical Epidemiology and Biostatistics Unit, Murdoch Childrens Research Institute, Royal Children’s Hospital, Parkville, VIC Australia; South Eastern Melbourne Medicare Local, Dandenong, VIC Australia; Southern Academic Primary Care Research Unit, Monash University, Dandenong, VIC Australia; Judith Lumley Centre, La Trobe University, Melbourne, VIC Australia; School of Population Health, University of Melbourne, Parkville, VIC Australia

**Keywords:** Quality improvement, Partnerships, Universal health services, Refugee families, Time series design, Process evaluation

## Abstract

**Background:**

The risk of poor maternal and perinatal outcomes in high-income countries such as Australia is greatest for those experiencing extreme social and economic disadvantage. Australian data show that women of refugee background have higher rates of stillbirth, fetal death in utero and perinatal mortality compared with Australian born women. Policy and health system responses to such inequities have been slow and poorly integrated. This protocol describes an innovative programme of quality improvement and reform in publically funded universal health services in Melbourne, Australia, that aims to address refugee maternal and child health inequalities.

**Methods/design:**

A partnership of 11 organisations spanning health services, government and research is working to achieve change in the way that maternity and early childhood health services support families of refugee background. The aims of the programme are to improve access to universal health care for families of refugee background and build organisational and system capacity to address modifiable risk factors for poor maternal and child health outcomes. Quality improvement initiatives are iterative, co-designed by partners and implemented using the Plan Do Study Act framework in four maternity hospitals and two local government maternal and child health services.

Bridging the Gap is designed as a multi-phase, quasi-experimental study. Evaluation methods include use of interrupted time series design to examine health service use and maternal and child health outcomes over a 3-year period of implementation. Process measures will examine refugee families’ experiences of specific initiatives and service providers’ views and experiences of innovation and change.

**Discussion:**

It is envisaged that the Bridging the Gap program will provide essential evidence to support service and policy innovation and knowledge about what it takes to implement sustainable improvements in the way that health services support vulnerable populations, within the constraints of existing resources.

## Background

The greatest potential to reduce health inequalities across the life course lies in giving children *a healthy start to life* [1,2]. A growing body of evidence shows that brain development is highly sensitive to external influences in utero and in early childhood, with potential for lifelong effects [3]. Infants born preterm, small for gestational age or with low birthweight have well-documented increased health risks in childhood, with recent evidence supporting continuing effects into later life [4]. Exposure to social adversity in early childhood is consistently associated with later life health problems including early onset of chronic conditions such as heart disease, diabetes and asthma, with effects compounded by the operation of an ‘inverse care law’ whereby people most in need of high quality health care are least likely to access timely care, including preventative health care [5-7].

Currently, the risk of poor maternal and perinatal outcomes in high-income countries such as Australia is greatest for Indigenous populations, refugee populations and for those experiencing extreme social and economic disadvantage [8,9]. Australian data show that women of likely refugee background have higher rates of stillbirth, fetal death in utero and perinatal mortality compared with Australian born women [10,11]. Refugee populations also have higher risk of a range of other physical, mental and social health problems related to experiences of extreme deprivation, trauma and stress and high rates of persistent disadvantage in the developed countries in which they settle [12-16].

These disparities warrant urgent action by health services. Unfortunately, health systems in high-income countries have not yet responded adequately to health inequalities affecting vulnerable populations. Policy and health service responses have been slow, poorly integrated with existing services, largely implemented without adequate service or community engagement and without consideration of the social determinants of health, despite calls to do so [17]. In a recent editorial about achieving health equity by design, Wong et al. argue that unless critical elements of health service redesign include a clear focus on specific communities at risk, meaningful data to understand local needs and priorities, a conviction to make progress, and ongoing assessment of health outcomes, then disparities in health will persist [18].

There are notable challenges in providing high quality care to families of refugee background. Concentrations of multi-layered risk associated with extreme social disadvantage at the critical life stage of having a baby require additional time and effort by care providers in order to attend to issues beyond screening and management of medical complications. Upon arrival in receiving countries and for many years post settlement, people who come as refugees or asylum seekers have to deal with multiple and often inter-related stressors associated with fleeing their country and establishing a new life [19]. Such stressors include physical and mental health issues, learning a new language, unemployment and underemployment, economic adversity, securing appropriate and affordable housing, social isolation, accessing services and trauma induced by refugee experiences [20-23]. The experiences and living conditions endured by many women of refugee background in their countries of origin and on their journey to their new country of settlement—unattended births, traumatic and unsafe abortions, use of unsterilised equipment, poor sanitation, female genital mutilation/cutting and high rates of fetal death in utero and infant mortality—contribute to the risk of obstetric complications and may cause women (and men) to be fearful and anxious about utilising maternity services [24]. The circumstances leading people to flee from their own country—experiences of persecution or having a well-founded fear of persecution—mean that many live with ongoing trauma symptoms, in addition to the accumulative stressors of settlement and loss or separation.

This paper describes the study protocol for an innovative programme of quality improvement initiatives and health system reform to address refugee maternal and child health inequalities—called Bridging the Gap—currently being implemented in public funded universal health services in two outer Melbourne regions which are major areas of settlement of people of refugee background. One of the major drivers for health services committing to involvement in the Bridging the Gap program was recognition of poor maternal and child health outcomes experienced by refugee families [10]. Leverage for change also came from an earlier study undertaken in 2012 documenting the significant challenges faced by health professionals caring for refugee families from Afghanistan in Melbourne’s south east, and multiple ways in which maternity care was failing to meet the needs of clients of refugee background [25]. This study involved interviews with Afghan women and men and with health professionals working in hospitals and community-based services. We uncovered considerable concern amongst health professionals and policy makers about gaps in service system capability for meeting the needs of families of refugee background [26].

### Partnerships for change

Eleven organisations have come together to form the Bridging the Gap Partnership and are working to achieve change in the way that maternity and early childhood services support families of refugee background. The original impetus for Bridging the Gap came from staff based at the Murdoch Childrens Research Institute (MCRI) and the Victorian Foundation for the Survivors of Torture (Foundation House). Foundation House provides services to people from refugee backgrounds who experienced torture or other traumatic events in countries of origin, assists health and other service providers to enhance their responsiveness to the needs of people of refugee backgrounds and engages in research to improve services for this population. Both organisations have a history of community and public-sector engagement and had recently worked together on the Afghan study [27].

MCRI and Foundation House jointly approached the other partner organisations to form the Bridging the Gap Partnership. The initial approach to the key stakeholders in each agency included a discussion of the proposed protocol, alignment with each organisation’s policy and service delivery agenda and the extent to which they were able to support the development and implementation of quality improvement and organisational change within existing resources. Several of the agencies approached to participate in Bridging the Gap had taken part in the stakeholder advisory group for the Afghan study [28]. The study had provided these agencies with qualitative evidence of the issues for refugee families in accessing maternity and early childhood health care and the challenges for health professionals in providing care for refugees [27]. Early on in discussions with these agencies, the idea of expanding the partnership to include organisations in Melbourne’s west was suggested. The west has a similarly large refugee population, and key stakeholders in the south-east identified that there would be benefits of sharing learnings about organisational change and quality improvement across agencies in the two regions. The partnership now comprises:

- two publically funded hospital networks (responsible for four maternity hospitals)
- two publically funded maternal and child health services (delivered in over 40 community-based clinics) in outer suburban municipalities
- two Commonwealth Government funded primary health care networks called ‘Medicare Locals’
- two state government departments
- the peak body for local government in Victoria
- the agencies responsible for initiating the partnership: MCRI and Foundation House (see Table 1 for a list of partner organisations).

**Table 1:** **Bridging the Gap partner organisations**

**Partner organisation**	**Description**
Murdoch Childrens Research Institute	Instigating partners: Healthy Mothers Healthy Families Research Group
Victorian Foundation for Survivors of Torture (Foundation House)	Instigating partners includes departments, policy and research, health sector development, Victorian Refugee Health Network
Monash health	Three maternity hospitals in Melbourne’s south-east
	Implementation site
Western health	One maternity hospital in Melbourne’s west
	Implementation site
City of Greater Dandenong	Local government maternal and child health service in Melbourne’s SE
	Implementation site
City of Wyndham	Local government maternal and child health service in Melbourne’s west
	Implementation site
Department of Health	Policy partner with policy and programme responsibilities for public maternity hospitals and refugee health
Department Education and Training (formerly Department of Education and Early Childhood Development)	Policy partner with policy, funding and programme responsibilities for maternal and child health (MCH) services across Victoria
South East Melbourne Medicare Local	Primary health care network in Melbourne’s south-eastern region
South West Melbourne Medicare Local	Primary health care network in Melbourne’s western region
Municipal Association of Victoria	Peak body for local government in Victoria; works in partnership with DDECD in planning, funding and supporting local government MCH service

**Figure 1:**
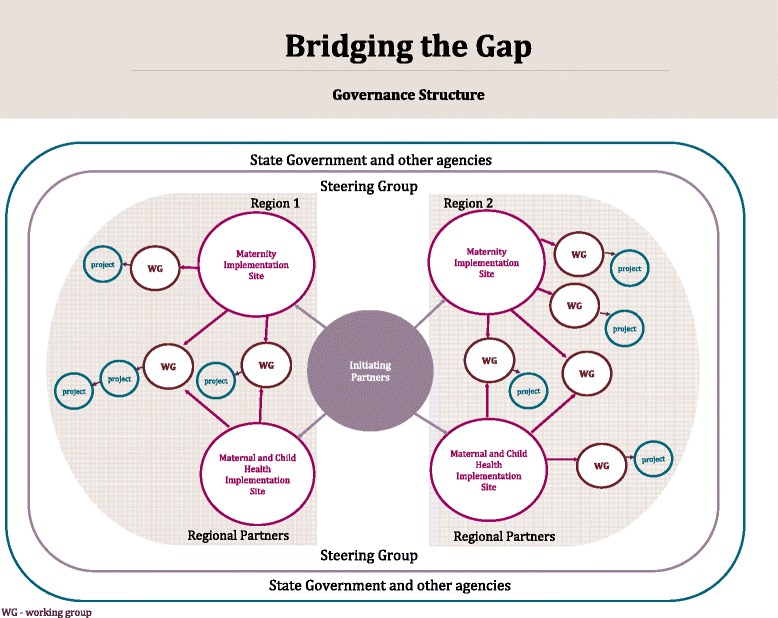
Bridging the Gap partnership and governance structure.

Partnership members came together on several occasions to develop a shared vision for the programme (Table 2). Governance arrangements (see Figure 1) were considered over a series of meetings of key stakeholders from partner organisations resulting in the formation of a steering group. The steering group meets bimonthly and has agreed to operate in accord with the principle of collaborative decision-making to support programme implementation and evaluation. Together, the partnership successfully sought nationally competitive research funding matching in-kind contributions from each partner organisation. The funding secured via a *Partnerships for better health* project grant from the Australian National Health and Medical Research Council supports programme facilitation and evaluation. Innovation and systems change in the health agencies is undertaken within their existing resources. The role of the research agency (MCRI) is to facilitate the work of the partnership and assist in the facilitation of the quality improvement working groups and measurement of process and outcomes.

**Table 2:** **Partnership vision for Bridging the Gap**

**Focus on**	** Vision**
Women & families who feel:	
	respected and treated with dignity
	engaged and confident
	safe
	their experience & needs are understood & valued
	they can communicate and be heard
Services that are:	
	welcoming
	responsive
	connected and well integrated
	seamless
	flexible
	family centred
	multidisciplinary
Workers who are:	
	knowledgeable
	sensitive
	culturally aware
	well supported

### Setting for programme implementation

The setting for programme implementation is four publicly funded hospitals providing care to pregnant women in two metropolitan regions and early childhood and primary care services offering support to families in the first year after having a baby. Pregnancy care in Australia can be accessed in either the public or private sector. Public pregnancy care is offered to women and families through public hospital antenatal clinics or in shared care arrangements between a community-based general practitioner and hospital antenatal clinic. Women booked as public patients have labour and birth care and care in the days following birth provided by rostered hospital staff.

The four public hospitals across the two metropolitan regions together account for around one fifth of all births in the state of Victoria. The three maternity hospitals of Monash Health serving Melbourne’s south-east and Sunshine Hospital serving the western suburbs are by far the largest providers of maternity care to refugee populations in Victoria. It is estimated that of the 14,000 women who give birth at these maternity services each year, around 10% are women of refugee background.

The Maternal and Child Health (MCH) Service in Victoria is a universal service for all families with children up to school age. All babies born in Victoria are automatically referred to the service through a legislated birth notification process. The service provides child health and developmental checks, screening and referral to additional services if required and assessment and support related to maternal health. Care is provided by maternal and child health nurses (with qualifications in nursing, midwifery and maternal and child health) and is organised around ten ‘Key Ages and Stages’ consultations, including a home visit shortly after birth and a further six visits in the infant’s first year [29].

The maternal and child health services of the City of Greater Dandenong in the south-east and the City of Wyndham in the west cater to diverse and rapidly growing refugee populations. In Greater Dandenong, 55% of the resident population were born in non-English speaking countries, with 8% settling in Australia in the past 2 years, more than double the settlement rate for greater Melbourne. City of Wyndham has experienced a 434% increase in the number of refugees living in the municipality over a 3-year period [30].

Whilst the participating regions have a track record of innovation, some short term projects to support refugee families have not been sustainable due to lack of recurrent funding. This is one of the drivers for Bridging the Gap. Health professionals at participating agencies have identified a pressing need for care better tailored to the needs of refugee communities. There was a clear commitment to tailoring of care in such a way that the process and outcomes of change are examined; initiatives found to be successful are embedded as standard practice and are sustained in the long term.

The implementation sites will introduce innovations in practice in parallel with each other in order to enhance learnings from implementation and facilitate knowledge exchange about organisational and system change—that is what works for whom, why, when and how.

### A case for change: evidence to inform quality improvement

Bridging the Gap is intended to be evidence informed and evidence generating. Although there is not a strong body of evidence to guide development of programme initiatives, several studies point to the importance of focusing efforts on improving access and engagement in care for vulnerable populations [31]. Analysis of routinely collected Victorian hospital and population-based data indicates that women of refugee background are less likely to attend the recommended number of antenatal check-ups and more likely to attend accident and emergency departments for obstetric complications [10]. Access to antenatal care in the first trimester of pregnancy and regular attendance at antenatal visits has a positive effect on maternal and child health [32-34]. Whilst there is no consensus about the optimal number of visits, there is evidence that inadequate antenatal care is related to worse pregnancy outcomes [35].

Similarly, families of refugee background are less likely to attend all scheduled early childhood health care visits. The platform of an early home visit followed by ‘Key Age and Stages Visits’ offered by the Victorian Maternal and Child Health Service in the first 12 months of the infant’s life is underpinned by strong evidence that primary health care support is essential to ensure early identification of problems affecting child growth and development [36]. A Victorian study identified significant barriers for refugee families that hamper access to MCH services, concluding that a system-orientated, culturally competent approach to service delivery would enhance access and the experiences of care [26].

Victorian maternity and early childhood health services are poorly integrated, operate independently of each other and are governed and funded by different government departments. Evidence from a number of studies indicates for services to respond to vulnerable families including those of refugee background; one critical factor is the close integration of primary and specialist health care services [37-39]. For example, the transition from hospital to community-based primary care such as general practitioners (who are either private practitioners or funded through national programmes) operates in a system separate again to that of maternity and early childhood health services. Recently released Victorian state government policy for the future direction of Victorian MCH service also identifies better integration of antenatal and postnatal services as a priority [40].

It is increasingly clear that failure to attend to the social aspects of families’ lives in pregnancy may hamper efforts to improve outcomes for vulnerable populations. The National Institute for Health and Clinical Excellence guidelines for antenatal care emphasise the importance of early and ongoing discussion of social factors and the tailoring of services to address the needs of asylum seeker and refugee populations [41]. This requires care providers to have a heightened awareness and understanding of the experience of forced migration and settlement in a new country and confidence implementing practical approaches to identifying and responding to complex needs, including issues such as limited health literacy, psychological distress, social isolation and family violence [42].

Efforts to improve access and targeted intervention strategies are hampered by the fact that women and children of refugee background are invisible in most health datasets [43-45]. Addressing this ascertainment challenge in these government-funded universal health services is therefore a starting point for any initiatives aiming to improve outcomes for the refugee population.

### Aims

The aims of the Bridging the Gap program are to implement and evaluate co-designed quality improvement strategies to:

- Improve access to universal health care for families of refugee background
- Build organisational and system capacity to identify and address modifiable risk factors for poor maternal and child health outcomes in refugee populations
- Develop a sustainable framework for ongoing quality improvement in responding to the needs of families from refugee background.

Whilst we expect that individual quality improvement interventions will address specific aims, we hypothesise that the Bridging the Gap program, with combined quality improvement initiatives and system reform, will result in measurable change in health and health care outcomes for families of refugee background and organisations that are more responsive to the needs of vulnerable families.

### Conceptual framework

Bridging the Gap’s design draws on Greenhalgh and colleague’s model of diffusion, dissemination and implementation of innovations in service organisations [46]. Several components of the model are integral to the programme and evaluation design. In particular, attention has been given to system antecedents for innovation. These include a receptive context for change encompassing leadership and vision of the partnership; system readiness for innovation including tension for change and dedicated in-kind resourcing; implementation processes including quality improvement decision-making devolved to frontline staff; and a participatory, action orientated implementation framework with feedback on process. Greenhalgh’s model provided a framework for the development of the Bridging the Gap protocol. Specifically, the model provided a framework and guide for the partner organisations to clarify expectations and develop a shared vision for how the partnership would operate. In practice, this has involved a commitment to shared leadership, collaborative decision-making and participatory approaches to co-designing quality improvement initiatives and facilitating organisational change.

Programme evaluation will assess the system components and implementation processes in addition to outer, socio-ecological contextual influences (i.e. socio-political climate); adoption of innovation over time; and communication and influence (i.e. support of programme champions).

## Methods

### Quality improvement initiatives

Bridging the Gap is designed under the premise that ‘If we keep on doing what we have been doing, we are going to keep on getting what we have been getting’ [47]. The development of quality improvement initiatives and their implementation is iterative. That is, the points of change are not predefined in the protocol. The partner organisations have identified several priority areas for improvement including better ascertainment of refugee background in data platforms to support targeted quality improvement, system improvements to support access and engagement of refugee families in care, options to enable families to access care close to home and the development of culturally appropriate information.

**Figure 2:**
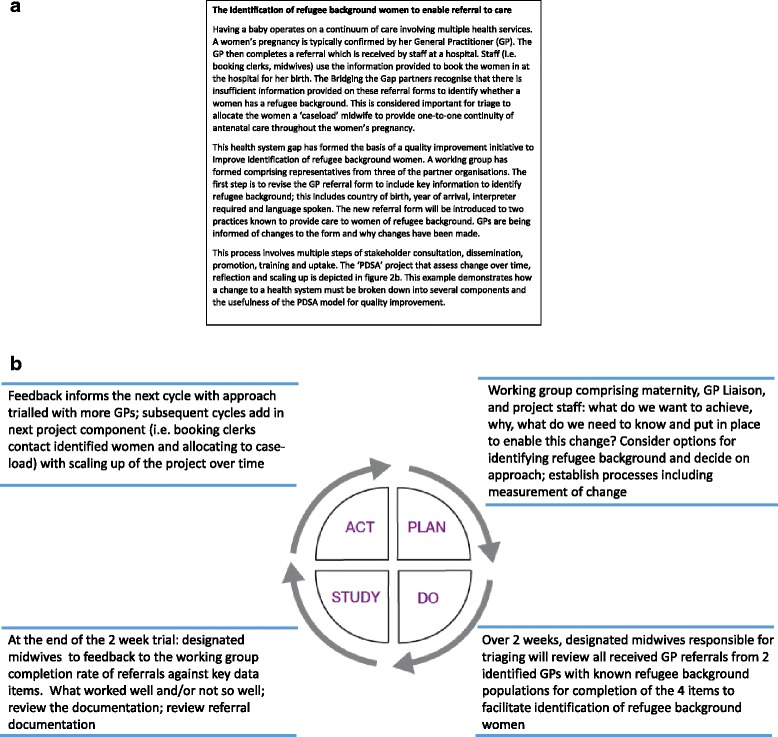
Bridging the Gap quality improvement example **(a)** and example quality improvement project and PDSA cycle **(b)**.

Some quality improvement projects will be inherently complex as they address multiple issues. This is illustrated in Figure 2a, b, an early example of partnership at work and the emerging complementary cycles of implementation involved in improving identification of refugee background in the maternity setting. Fundamental to implementation of quality improvement initiatives is workforce training across participating maternity hospitals and maternal and child health services. The partnership recognised early on that this would be central to enhancing staff’s understanding of the refugee experience and working with families who have experienced torture and trauma. The need for training, designed and delivered jointly by partnership organisations, has emerged as a critical building block to assist staff in working differently in caring for families of refugee background.

#### Implementation: making change happen

In the context of the Bridging the Gap program, co-design encapsulates the work of the partnership as a design and implementation collaborative. We agreed on the framework for implementation including collaborative decision-making at a regional level and working together with health care providers in project planning and implementation cycles.

Quality improvement interventions will be implemented over a period of 3 years (2014–2016).To facilitate design, implementation and fine-tuning of the interventions, the Plan, Do, Study, Act (PDSA) framework is being utilised [48] (see example at Figure 2b). The partnership adopted the PDSA framework as a pragmatic method for implementing and testing changes through small rapid cycles of improvement, with flexibility to adapt change according to feedback and engage stakeholders in each PDSA cycle [49,50].

The participation of clinical staff and managers is pivotal to each aspect of bringing about change including the design of quality improvement strategies, implementation and the review of progress. This in turn provides a feedback loop to support ongoing engagement and intervention refinement. The role of the research agency (MCRI) is to synthesise the available evidence, provide support for intervention development and together with stakeholders from the partnership, analyse and translate the findings in a continuous cycle of quality improvement and refinement. In practice, a major part of the work done by the research agency staff involves bringing people together and facilitating the co-design, implementation and evaluation of Bridging the Gap initiatives.

The partnership is committed to sustainability of quality improvement with built-in processes to ensure potential impacts of context and innovation, and the capacity of the organisation to sustain initiatives are considered throughout the life of the programme. Context mapping and attention to innovation fidelity-maintenance strategies will include workforce training and audit and feedback through the PDSA cycles.

### Evaluation study design and data collection

**Figure 3:**
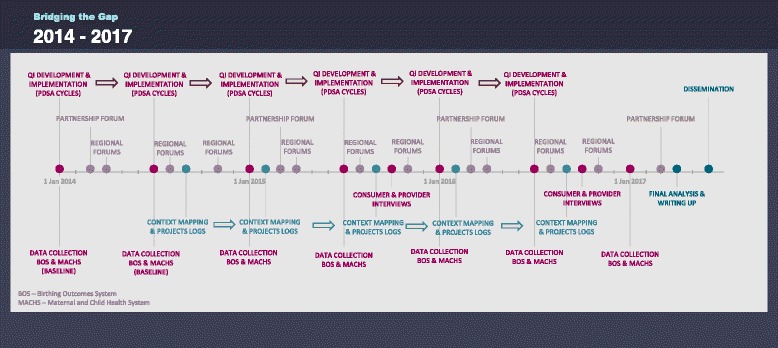
Timeline of quality improvement interventions and evaluation.

Bridging the Gap is designed as multi-phase, quasi-experimental study. Evaluation methods include use of interrupted time series design to examine health service use and maternal and child health outcomes over the period of implementation of quality improvement interventions. This will be complemented by detailed process evaluation. See Figure 3 for timeline of programme implementation and concurrent evaluation.

#### Time series design: monitoring health service usage and maternal and child health outcomes

The primary outcomes for the study are (1) the proportion of women of refugee background booking for birth at participating hospitals attending seven or more antenatal visits and (2) the proportion of families of refugee background registering with participating MCH services attending all Key Ages and Stages MCH visits in the first 12 months postpartum.

Secondary outcomes include measures of organisation and system performance and maternal and child health outcomes for refugee families at the participating sites. These are detailed in Table 3.

**Table 3:** **Perinatal and maternal and child health outcome data collected six monthly (2014–2017)**

**Maternity hospitals**	**Maternal and Child Health services**
Socio-demographic and obstetric characteristics	Socio-demographic and obstetric characteristics
Maternal country of birth	Maternal & paternal country of birth
Year of arrival in Australia	Year of arrival in Australia
Maternal date of birth	Maternal date of birth
Relationship status	Infant’s date of birth/age
Infant’s date of birth	Parity, plurality, gravidity
Parity, plurality, gravidity	Place of birth
Pre-existing conditions (e.g. female genital cutting, diabetes)	
Primary outcome	Primary outcome
Completion of seven or more antenatal visits	Attendance at the first seven Key Ages and Stages visits
Secondary outcomes	Secondary outcomes
Antenatal visit in the first trimester (<14 weeks)	Registrations at MCH via birth notifications
Completion of tests for gestational diabetes at <30 weeks’ gestation; outcome of testing	Completion of immunisations to 12 months
Completing of other screening tests: anaemia, urinary tract infection, hepatitis B, syphilis, vitamin D	Identification of developmental or other major health issue
Discussion of psychosocial issues with hospital staff during pregnancy (i.e. emotional wellbeing; family violence)	Referral of infant to GP, paediatrician, allied health services
Induction of labour	Breastfeeding at 6 weeks and 6 months
Infant born before arrival at hospital (unplanned)	Maternal health and wellbeing assessment completed; counselling or referral for mental health issues
Epidural	Family violence screen completed; counselling or referral for family violence
Mode of birth	
Perineal trauma	
Postpartum haemorrhage	
Infant birthweight; gestational age at birth	
Admission to special care nursery or neonatal intensive care unit	
Stillbirth	
Breastfeeding in hospital	
Length of hospital stay following birth	

Routinely collected perinatal and maternal and child health data provide a repository of information on all births and maternal and child health outcomes in Victoria. Data will be accessed from two independent databases: the Birthing Outcome System (BOS) in maternity and the Maternal and Child Health data system. Implementation sites will extract data for a 12-month period prior to commencement of initiatives (baseline) and at 6 monthly intervals over the 3 years of intervention implementation. Data will be made available to the research team at MCRI in non-identifiable format. Audit of medical records at baseline will be used to validate data on the number of pregnancy check-ups recorded in the Birthing Outcome System at the participating hospitals and assess potential variability in how data are recorded.

#### Sample and study power

Approximately 14,000 women give birth in the four hospitals each year. Using maternal country of birth as a single data item to ascertain refugee status is problematic, as it is not possible to distinguish women coming to Australia as humanitarian entrants from women coming via other pathways. At the time of developing the study protocol, we conservatively estimated that around 700 women birthing at one of the four hospitals were of refugee background. An additional constraint at the time of conducting sample calculations was the inability to access data from the BOS for pre-specified primary outcomes. For this reason, initial sample calculations were based on data from a Victorian population-based survey of women giving birth in 2007. Data were obtained for a sub-sample of disadvantaged mothers taking part in this survey, selected based on their experience of stressful events and social health issues, matching the complex social circumstances as women of refugee background [51,52]. We considered that the multi-faceted initiatives would be successful and clinically significant if the proportion of the ‘refugee-like’ group not receiving seven or more antenatal visits was halved from 30% to 15%. Several factors complicate sample size calculations. First, mothers will be clustered within hospitals, decreasing statistical power [53]. Second, because of the time series design, the study will have increased statistical power (variance reduction) compared to studies where the outcome is only measured at one point in time [54]. Third, period effects will need to be adjusted for in analysis, including change in the proportion of refugee mothers who have arrived in Australia in the previous 6 months. The adjustment for potential confounding will slightly reduce statistical power [55].

Preliminary data from Maternal and Child Health Service suggests that for a ‘refugee-like’ group, around 45% will not complete the first seven Key Ages and Stages visits. The threshold for success would be to decrease this to 15%, the average percentage for the entire population.

Based on a sample size of 350 in each 6-month period, the study will have 88% power to detect a halving of the antenatal primary outcome (<7 pregnancy visits) from 30% to 15% (alpha = 0.05, intraclass correlation 0.05). For reducing the number of families who do not complete the schedule of KAS visits from 45% to 15%, the power will be 93%, ranging from 82% to 98%, depending on the values for the intraclass correlation, correlation over time and correlation between explanatory variables.

#### Planned analysis

A time series comparison of proportions will be used to assess trends in primary and secondary outcomes at 6 monthly intervals, with adjustment for potential period effects. Potential period effects could include for example, changes in number of refugee mothers arriving in Australia in the 6-month period of data collection or a major change in policy affecting the access of families of refugee background to services. Multilevel regression models will be used to account for clustering of mothers within hospitals and local government areas (LGAs) and correlation of observations across hospitals and LGAs over time and potential period effects.

#### Process evaluation: refugee families’ experiences of services

The experience of families will be explored via focus groups at two time points: 18 months into implementation and at the end of the implementation period. Women from countries representing the largest refugee population groups attending the implementation sites (from Afghanistan, Sudan and Burma) will be invited to participate. Approximately 100 women will participate in groups facilitated by bicultural research assistants at around four to six months postpartum. Purposive recruitment will take place via community playgroups and at scheduled health visits, for example, immunisation clinics. Focus group questions will cover experiences of specific aspects of maternity and MCH care including women’s experiences of care under the new quality improvement initiatives. For example, Karen, women from Burma attending new community-based pregnancy sessions will be asked about accessing the service and the responsiveness of care to their individual needs. Questions in focus groups will also include first contact with services, use and experience of language services, responsiveness to emotional and social health issues, referral pathways, provision of information and advice and transition from hospital to community-based services. Focus group data will be analysed thematically [56].

#### Process evaluation: service level experience of organisation change

Assessment of organisational change will be undertaken considering individual components of innovation and the interaction between components, with particular reference to local context, setting and timing [57,49]. Ongoing mapping of context and process, programme event logs and interviews with staff from the programme implementation sites will provide critical information on the nature of the organisation context (size, designated resources, leadership and champions of change); each organisation’s readiness for developing and implementing quality improvement initiatives; the characteristics, views and experiences of providers about the process of change and adoption of innovation; the nature of communication and influence of the improvement project; and the nature of the external context and how this impacts on the assimilation process. Critical to sustainability is the workforce and the interpersonal processes that champion innovation. Mapping includes documentation of workforce characteristics and stability.

Interviews and focus groups will be conducted with clinicians, service managers and other stakeholders affiliated with the interventions (e.g. staff from language services, social workers, general practitioners) using a narrative approach to ‘telling the story’ of organisational change [49]. Around 70 staff and stakeholders will participate in interviews and focus groups conducted at two time points during implementation.

The facilitation team at MCRI is responsible for programme mapping. A programme log will document phases of development and implementation. Information on internal and external influences that may impact on the organisation and delivery of care will be recorded, and mapping will include a repository and synthesis of service plans and reports.

#### Ethical approval

The time series design for the use of routinely collected perinatal and maternal and child health data has been approved by the Human Research Ethics Committees (HREC) of the Royal Children’s Hospital, Monash Health, Western Health, and the Department of Education and Training (formerly Department of Education and Early Childhood Development). HREC approval for the process evaluation is currently under consideration. The interventions and associated PDSA cycles of implementation are registered with the research and ethics offices of the Monash Health and Western Health as quality assurance projects, complying with the National Health and Medical Research Council’s report of ethical considerations for quality assurance [58].

#### Study status

The study will be completed in 2017. At the time of writing, four quality improvement initiatives and staff training are underway, the first wave of time series data retrieval is complete, and planning for process evaluation involving families of refugee background and health care providers in 2015 and 2016 has commenced. A programme website is auspiced by MCRI [59]. Partners in the western region formally launched Bridging the Gap in November 2014 and followed the launch with professional development activities related to working with refugee families.

## Discussion

Bridging the Gap is an ambitious multi-faceted programme to address social inequalities in refugee maternal and child health outcomes. Several factors make it unique. The 11 partner organisations—spanning research, refugee programmes health services, primary health care networks and local and state government—have agreed to work together to effect change. Each partner organisation has committed cash or in-kind resources to the programme and has documented the proposed benefits of Bridging the Gap for their organisation and constituency. As we near the end of the first full year of programme implementation, organisations have far exceeded their projected in-kind contributions, with substantial investment of time, energy and enthusiasm for ‘doing things differently’ to improve care and outcomes for refugee families.

The Bridging the Gap partnership also brings together two different sectors of the Victorian health system (maternity and maternal and child health) which historically have operated independently. Our partner organisations have recognised significant collaborative advantage in developing and trying out new ways to work together in caring for vulnerable families [60]. Recognition that there is a need to move beyond a silo approach to health care and work towards integrated systems for improving health outcomes has been an additional impetus for collaboration.

Attention has been paid to best positioning partnership success, recognising what it takes to enable collaborative effort. Key components of successful partnership include having a shared vision, shared decision-making and leadership [5]. The vision for Bridging the Gap was developed by the partnership group. The partner organisations have also developed principles for the way that we work together, and these are presented routinely at the many meetings of the steering group, regional partners and project working groups as a reminder of our agreements. The layers of programme co-leadership are defined, with leaders of quality improvement initiatives emerging as training and project implementation gain traction.

Engineering change within complex health systems is notably challenging, sustaining change inherently difficult. Whilst the protocol has been developed to optimise sustainable change and the partnership continues to draw on the learnings of others in positioning Bridging the Gap for success, we are mindful of limitations. The maternity implementation sites are all situated in large general hospitals with competing demands for acute care resources. Other pressures on the health agencies include rising birth rates and changing demographics in the participating regions coupled with periodic organisational restructuring and fluctuations in workforce (i.e. shortage of doctors and midwives). Oscillating national and state asylum seeker health service eligibility policies compound difficulties for services in meeting the needs of women and families newly arrived in Australia. Pressures and demand can be either a lever for sustainable change or a barrier.

Facilitation of the partnership and project working groups requires considerable resourcing. National and partner organisation funding supports programme evaluation including a part-time data analyst and one part-time programme facilitator at MCRI. All other programme facilitation by study investigators based at MCRI and FH is undertaken as an in-kind contribution from these organisations, with to date only a small amount of funding contributed by partner agencies and/or secured from other sources to contribute to salary costs.

Comprehensive multi-method evaluation will enable careful recording of the factors that may limit organisational change over the 3-year period of implementation. The use of routinely collected data to monitor outcomes over time lessens the burden on staff to collect data for evaluation purposes. An additional strength of the evaluation is that the time series design is complemented by process evaluation providing a deeper understanding of the ‘how’, ‘why’ and ‘at what cost’ intervention outcomes are achieved, including sustainability and potential transferability [61].

The first year has seen significant progress in collaborative efforts to identify families of refugee background in agency data, building workforce capacity to work with refugee families through training and the implementation of the first quality improvement projects. Challenges include the time taken to establish relationships with staff and identify champions within an environment of competing demands; the level of MCRI facilitation required within the limitations of funding; ‘external influences’ including a change of government and restructuring within several partner organisations; and the protracted time required to incorporate new data items into health service data systems.

There are encouraging early signs that the programme may well be influencing policy, evidenced in several recently released policy initiatives from partner organisations. The Victorian Department of Health’s refugee and asylum seeker action plan articulates new approaches to improving the physical and mental health of refugees across the state [21]. Bridging the Gap is profiled as a best practice case study of partnerships to effect change. The Department of Education and Training’s new blueprint for the future direction of the maternal and child health service builds on the Marmot review emphasising proportionate universalism [1]. This government department plan highlights the need to work across the continuum of pregnancy and early childhood care and engage vulnerable families in a more culturally inclusive service for refugee families [40].

In conclusion, Bridging the Gap is a set of multi-faceted and multi-organisational set of quality improvement and system reform initiatives aimed at improving health and health care outcomes for families of refugee background. The programme is partnership-driven and co-designed by providers, policy makers and researchers to maximise ownership, feasibility and sustainability of new ways of providing health care within maternity and early childhood health sectors. Process and outcome measures will provide critical feedback throughout the 4 years of implementation. The programme will provide essential evidence to support service and policy innovation and knowledge about what it takes within the constraints of existing resources to implement sustainable improvements in the way that health services support vulnerable populations.
